# Maternal vaccines: ten considerations for vaccine introduction and scale-up in low- and middle-income countries from the maternal, newborn, child, and adolescent health perspective

**DOI:** 10.1080/21645515.2020.1865047

**Published:** 2021-03-24

**Authors:** Sadaf Khan, Jessica A. Fleming, Cyril M. Engmann

**Affiliations:** a Maternal, Newborn, Child Health, & Nutrition, PATH, Seattle, WA, USA; b Center for Vaccine Innovation and Access, PATH, Seattle, WA, USA; c Departments of Pediatrics & Global Health, USA; d Schools of Medicine & Public Health University of Washington, Seattle, WA, USA; e Seattle Children’s Hospital, Seattle, WA, USA

**Keywords:** Maternal immunization, maternal health, integration, neonatal morbidity and mortality, pregnant women, antenatal care

## Abstract

Despite significant reduction in childhood mortality, infant – particularly neonatal – mortality continues to be unacceptably high. A substantial proportion of these deaths could be averted by vaccinating mothers during pregnancy (maternal immunization). However, in order to realize the full life-saving potential of maternal immunization, it is important to develop clear introduction and delivery strategies for maternal vaccines. This will necessitate close collaboration between maternal health and immunization stakeholders. This article examines key considerations and areas for action to support successful and sustainable introduction and scale-up of maternal immunization, from the perspective of maternal, newborn, child, and adolescent health stakeholders.

Vaccines are one of the most transformative public health innovations of the last century. The World Health Organization (WHO) estimates that between 2010 and 2015 alone, 10 million lives were saved due to immunization^1^ – many of them, children under five. While there have been notable declines in childhood mortality, similar reductions in infant mortality have lagged. An estimated 4.5 million children die before their first birthday each year, with nearly two-thirds of these deaths occurring in the neonatal period.^2^ An immunization strategy that considers the specific needs of young infant populations can help avert substantial mortality. Maternal immunization (MI), the practice of vaccinating mothers during pregnancy, is just such a strategy. MI not only protects the mother but enables protective antibodies to be transferred to her child in utero and via breast milk. This protects infants in the early, most vulnerable months following birth, when their own immune systems are immature and unable to confer protection. MI has a safe and effective track record against several diseases, including tetanus, influenza, and pertussis. One successful program, the Maternal and Neonatal Tetanus Elimination (MNTE) initiative, is often considered the “poster child” for MI success. WHO’s MNTE efforts to vaccinate pregnant women in low- and middle-income countries (LMICs) against tetanus have been largely responsible for the 88% decline in global neonatal tetanus deaths (from 200,000 in 2000 to 25,000 in 2018), leaving only 12 countries yet to eliminate the disease. However, MNTE’s high vaccination coverage was not achieved by solely relying on routine immunization services; rather, it also utilized extensive supplementary immunization activities to reach target populations.^3^ One lesson from the MNTE experience is that new approaches will likely be needed to achieve desired MI coverage levels where strong and equitable health systems do not exist.^4^

Beyond tetanus, there has been limited introduction and scale-up of vaccines recommended for pregnant women, particularly in low-resource settings.^5^ With several new maternal vaccines on the horizon (notably against respiratory syncytial virus and Group B Streptococcus), and the need to efficiently and effectively deploy epidemic vaccines to pregnant women against Zika, Ebola, and likely COVID-19, there is wide acknowledgment that the delivery of routine maternal vaccines will require approaches tailored to this population.

The Expanded Programme on Immunization (EPI) has had significant success in reaching children under age five, improving coverage from less than 5% to 86% for six target diseases^1^ from its inception in 1974 to 2017.^6^ The program delivers and reports on tens of millions of vaccines provided each year. Expanding this high coverage to pregnant women by relying on current traditional immunization services is likely to face challenges, as the system was primarily created to identify and reach young children. The antenatal care (ANC) platform, on the other hand, has a broad reach and access to pregnant women; more than 80% of women globally receive at least one ANC visit. An optimal approach to MI delivery will leverage the strengths of both the EPI and ANC programs and require strong coordination between immunization and maternal, neonatal, child, and adolescent health (MNCAH) stakeholders to reach the target population. As new maternal vaccines are introduced to countries, it will be important to develop clear introduction and delivery strategies that include common principles and approaches globally, while allowing flexibility to accommodate country context, including cultural, political, and social factors.

Viewing MI delivery from the perspective of MNCAH stakeholders, we identified 10 key areas for action, outlined below. We draw these observations from our work in coordinating the Advancing Maternal Immunization collaboration^7^ and participating as stakeholders in recent global efforts^8,9^ that brought the MNCAH and immunization communities together discuss strategies to strengthen the delivery of maternal vaccines.

## 1. Shifting the paradigm from a vaccine-specific delivery platform to a service delivery platform that integrates ANC and the EPI

The nature of maternal vaccines and their target population brings unique opportunities and challenges not encountered when deploying routine infant vaccines, and will greatly benefit from strong and effective integration and partnership between the EPI and MNCAH so MI efforts succeed and remain sustainable. While ANC service delivery has effective reach, staff may have less experience delivering vaccines and there are concerns about a potential negative impact on other services delivered through this platform. This suggests a need for moving from a vaccine-focused approach to a service delivery platform approach for MI, focused on the care women receive during pregnancy. This will leverage the ability of ANC services to identify and reach pregnant women and the EPI’s streamlined planning, logistics, and reporting mechanisms. Linking the MI agenda to the continuum of care and integrating MI into the life-course approach^10^ may also help engage a wider swathe of stakeholders across reproductive, maternal, newborn, child, and adolescent health.

## 2. Engaging key MNCAH stakeholders early in planning, introduction, and rollout

The complexity and multifactorial nature of maternal health pose unique advocacy needs for building consensus around introducing any new service to pregnant women, including vaccines. In addition to the traditional immunization policy experts, we need to include maternal health-care stakeholders working in policy and service delivery. These include professional societies (midwifery and obstetrics and gynecology), health-care providers, women’s rights advocates, and pregnant women themselves. It will be critical to engage these groups in early conversations around decision-making to incorporate their perspectives into introduction planning and program rollout.

## 3. Making the case for MI

Given multiple competing priorities in the MNCAH space, the case for “why MI” needs to be clearly and effectively communicated to support stakeholder awareness, engagement, and support. It will be critical to package evidence on disease burden, vaccine effectiveness, overall health impact, and the cost-benefit of MI in a manner that is appropriate for a range of MNCAH stakeholders, including financial and budgetary managers.

## 4. Including MI in efforts focused on optimizing the ANC platform

In 2016, WHO rolled out new ANC recommendations with a focus on women-centric care.^11^ As countries work to adapt and systematically roll out these guidelines, there is a window of opportunity to strengthen ANC systems and the package of services delivered overall, including documenting evidence on successful introduction and implementation of context-specific ANC services in LMICs. We recommend that MI introduction be considered within the overall context of improvements to ANC, rather than as a focused, stand-alone activity.

## 5. Learning from past integration efforts

There is a significant concern among MNCAH stakeholders about the overall impact on a new service such as additional vaccines will have on the existing systems, logistics, and workflows of an already over-burdened ANC platform. However, integration of formerly vertical services is not a new concept for ANC; services such as malaria and HIV prevention and treatment have been successfully integrated into ANC,^12^ which demonstrates the potential for greater sustainability and program efficiency when broader health-care systems are leveraged within vertical programs. Documenting these experiences and incorporating lessons learned into introduction planning will help streamline future introduction and integration efforts to address provider concerns and minimize implementation disruptions.

## 6. Identifying cross-program “win-wins”

There has been movement over the last 5 y in the MNCAH commodities space to leverage the EPI cold chain to ensure the quality of maternal medicines.^13^ Potential integration of MI into ANC may provide additional supplemental benefits and opportunities for leveraging EPI systems to implement recommendations around cold chain for heat-sensitive MNCAH medicines. Additionally, expanding the range of services available via the ANC platform may help improve perceptions of quality of care, which in turn could improve service uptake and reduce drop-off of follow-up care. From an immunization perspective, building ANC provider capacity to provide vaccines may reduce missed opportunities for immunization. Ultimately, the whole would be greater than the sum of its parts, with both programs benefiting from working together.

## 7. Equipping health-care providers with the knowledge and skills to become MI champions

Health-care providers have been acknowledged as extremely influential in shaping women’s decisions around receiving health-care services, including vaccines.^14^ However, providers may often be restricted in their ability to effectively counsel and motivate women to take up maternal vaccines, due to limitations in their knowledge and counseling abilities around maternal immunization. Addressing provider concerns, building capacity to effectively counsel patients, and providing appropriate knowledge resources to do so will help create “MI champions” among providers, further improving the uptake of maternal vaccines. Efforts should also focus on building the capacity of women’s groups and grassroots organizations to create demand through effective advocacy and communication approaches.

## 8. Supporting a collaborative exploratory research agenda

Recent research efforts and gatherings have brought together MNCAH and immunization stakeholders and demonstrated strong concordance among identified research priorities between the groups. Key exploratory questions and evidence needs for introduction planning are common to any new vaccine delivered via an integrated platform. However, given the range of stakeholders working in MI, there is a need to streamline efforts, leverage sufficient resources, and support coordination and harmonization among stakeholders. This will necessitate building on current ANC delivery practices and being responsive to the perceived impact of MI on service delivery, women’s care-seeking behaviors, community perceptions, and follow-up care in pregnancy. Other relevant information needs include the timing, nature, and quality of ANC, including strategies and mechanisms for follow-up with women not seeking care or missing subsequent visits. For vaccines intended to be administered within specific time windows, there is particular interest in collating information on the number and timing of ANC visits in order to identify timing beyond the first visit and frequency beyond the fourth visit, which are not currently captured in large surveys. Additional research needs include cost-effectiveness and cost-benefit analyses to support the economic argument for vaccine introduction.

## 9. Developing a context-specific, MNCAH-centric implementation science agenda

Among key areas for evidence generation post-vaccine introduction are identifying the most feasible models for the appropriate management and effective coordination between the EPI and MNCAH, and estimating the impact of MI introduction on overall ANC service delivery, workflows, and quality of care. Demand-side impacts include assessing improvements in ANC coverage, uptake of follow-up care, and perceptions of quality of care. In addition, individual countries may have unique needs that should guide their implementation agendas.

## 10. Strengthening monitoring systems for birth outcomes

Comprehensive data on background rates of pregnancy outcomes, including adverse outcomes in LMICs, would provide data to support maternal vaccine safety outside of clinical trial settings. This could avert issues of vaccine hesitancy attributed to adverse outcomes that are temporally associated but not vaccine related. To provide the necessary background data, robust surveillance systems for birth outcomes should be linked to immunization monitoring and surveillance systems in LMICs to track and report pregnancy and birth outcomes, vaccine coverage, and adverse events following immunization.

## 11. Conclusion

As we work toward optimizing the delivery of MI in LMICs, we have the unique opportunity to build on the EPI’s successes in preventing morbidity and mortality in children while simultaneously improving the health of pregnant women and their infants. Opening the EPI’s door to MNCAH colleagues and offering a seat at the table as full partners in MI now will go a long way toward ensuring that current and future maternal vaccines are delivered effectively, efficiently, and equitably to pregnant women. The opportunities for collaboration between MNCAH and immunization programs are natural and abundant. When colleagues across disciplines work together, we will avert morbidity and mortality and maximize positive health outcomes saving a significant number of young lives every year. With nearly a billion more births expected over the next decade, expanding our disease control armamentarium is more urgent than ever.
